# Changes and transcriptome regulation of endogenous hormones during somatic embryogenesis in *Ormosia henryi* Prain

**DOI:** 10.3389/fpls.2023.1121259

**Published:** 2023-04-03

**Authors:** Gaoyin Wu, Xiaoli Wei, Xiao Wang, Yi Wei

**Affiliations:** ^1^ College of Forestry, Guizhou University, Guiyang, Guizhou, China; ^2^ College of Life Science, Guizhou Normal University, Guiyang, Guizhou, China

**Keywords:** *Ormosia henryi*, somatic embryogenesis, differentially expressed genes, endogenous hormone, transcription factors

## Abstract

**Introduction:**

Ormosia henryi is a rare and endangered plant growing in southern China. Somatic embryo culture is an effective measure for the rapid propagation of O. henryi. It has not been reported how regulatory genes induce somatic embryogenesis by regulating endogenous hormone changes during the process of somatic embryogenesis in O. henryi.

**Methods:**

In this study, we analysed the endogenous hormone levels and transcriptome data of nonembryogenic callus (NEC), embryogenic callus (EC), globular embryo (GE) and cotyledon embryo (CE) in O. henryi.

**Results:**

The results showed that the indole-3-acetic acid (IAA) content was higher and the cytokinins (CKs) content was lower in EC than in NEC, and the gibberellins (GAs) and abscisic acid (ABA) contents were significantly higher in NEC than in EC. The contents of IAA, CKs, GAs and ABA increased significantly with EC development. The expression patterns of differentially expressed genes (DEGs) involved in the biosynthesis and signal transduction of auxin (AUX) (YUCCA and SAUR), CKs (B-ARR), GAs (GA3ox, GA20ox, GID1 and DELLA) and ABA (ZEP, ABA2, AAO3, CYP97A3, PYL and ABF) were consistent with the levels of endogenous hormones during somatic embryogenesis (SE). In this study, 316 different transcription factors (TFs) regulating phytohormones were detected during SE. AUX/IAA were downregulated in the process of EC formation and GE differentiation into CE, but other TFs were upregulated and downregulated.

**Conclusion:**

Therefore, we believe that relatively high IAA content and low CKs, GAs and ABA contents contribute to EC formation. The differential expression of AUX, CKs, GAs and ABA biosynthesis and signal transduction genes affected the endogenous hormone levels at different stages of SE in O. henryi. The downregulated expression of AUX/IAA inhibited NEC induction, promoted EC formation and GE differentiation into CE.

## Introduction

*Ormosia henryi* Prain is an evergreen tree belonging to *Ormosia* and is one of the precious timber trees in China. Due to the scarcity of its wild resources, breeding and protecting them are urgent issues. Somatic embryogenesis (SE), which is the process of dedifferentiation of somatic cells into somatic embryos and further development into a large number of embryoid bodies, is an efficient method of asexual reproduction (Cui and Dai, 2000). SE is widely used in the propagation of rare species and the preservation of germplasm resources. The author’s previous study established an SE regeneration system for *O. henryi* (Wu et al., 2020) and revealed the physiological and biochemical mechanism of EC formation, EC development and differentiation in the SE process, as well as the changes in cell tissue structure in different periods (Wu et al., 2021). However, the changes in endogenous hormones and the molecular mechanisms by which these changes are regulated during SE have not been reported.

Previous studies have shown that plant endogenous hormones play important roles in the development and morphogenesis of explants. Auxin and cytokinin levels affected the dedifferentiation and redifferentiation of plant cells and determined tissue regeneration and cell fate (Ikeuchi et al., 2019). Gibberellin and abscisic acid are crucial for the maturation and germination of somatic embryos (Khan et al., 2012; Li and Li, 2019). All these endogenous hormone levels are regulated and influenced by genes related to their biosynthesis and signal transduction. The *YUCCA* gene, as a key enzyme in auxin biosynthesis, is necessary for stem cell formation in *Arabidopsis thaliana* (Bai et al., 2013), EC formation in *Gossypium hirsutum* (Zheng et al., 2014), and somatic embryo development and maturation in *Musa* spp. (Enríquez-Valencia et al., 2019). Hwang et al. (2012) reported that *B-ARRs*, as key components involved in Arabidopsis callus induction, played an important role in shoot apical meristem regulation. The differential expression of *GA3ox*, *GA20ox*, *AAO3* and *PYL* genes related to gibberellin and abscisic acid was of great significance in the development of somatic embryos for *G. hirsutum* (Cheng, 2016; Jie, 2016) and *Medicago truncatula* (Rose, 2019). In addition, *AUX/IAA* and *ARF* genes, as auxin signal transduction genes, have a high regulatory effect on SE development, but they have different expression patterns in the SE process for different plants (Liu et al., 2019; Quintana-Escobar et al., 2019; Chen et al., 2020). Therefore, it was necessary to reveal the mechanism of endogenous hormones regulating SE in *O. henryi* by studying the changes in endogenous hormones and analysing transcriptome data.

In this study, NEC, EC, GE and CE in the SE process for *O. henryi* were used as test materials to detect endogenous hormone levels by LC−MS/MS and analyse RNA-Seq data. Aim at revealing the changes of endogenous hormone levels during SE in *O. henryi*, exploring the molecular mechanisms of the formation of NEC and EC and the differentiation of GE into CE, providing a theoretical basis for optimizing the SE system of *O. henryi.*

## Materials and methods

### Plant materials

**Figure 1:**
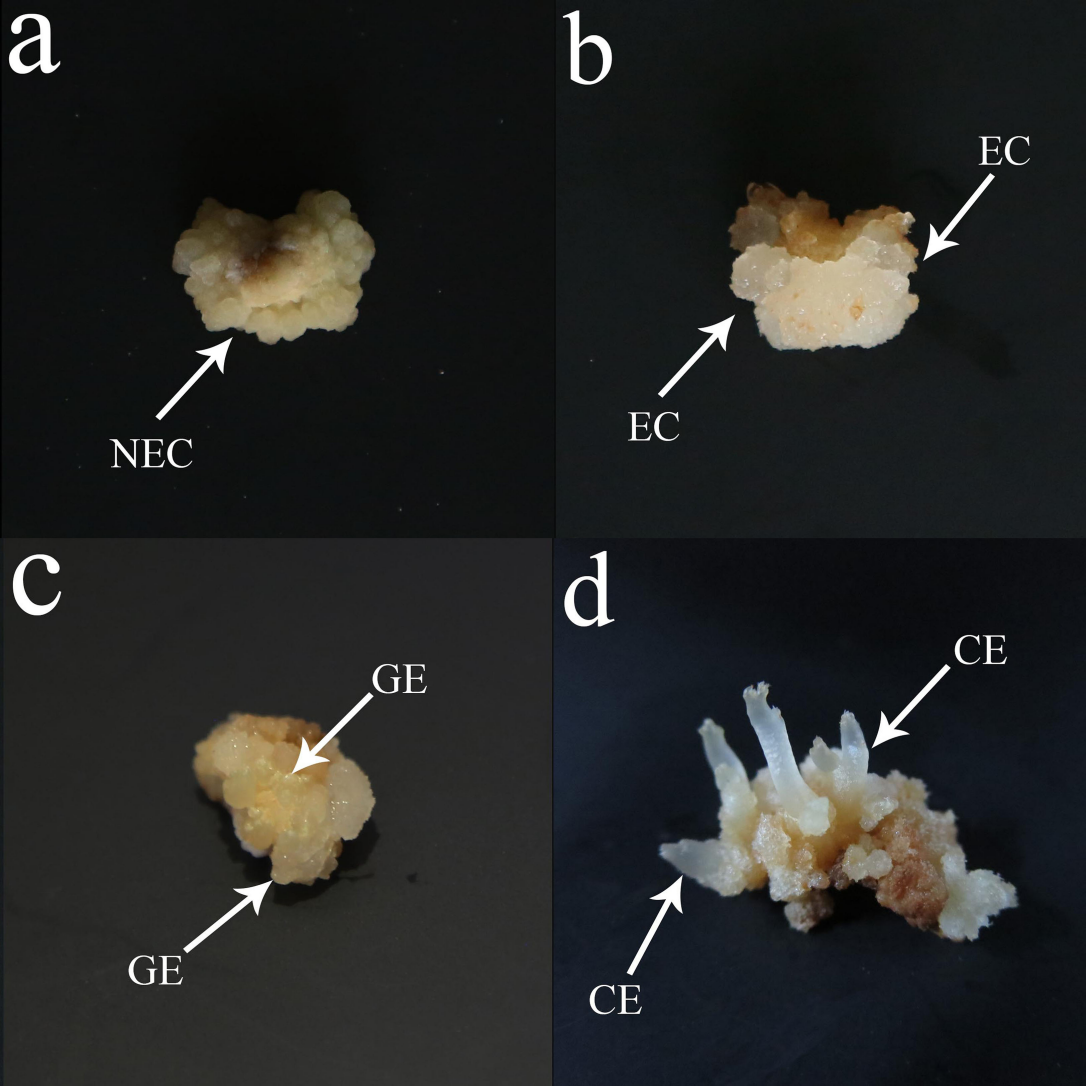
Somatic embryogenesis in O. henryi. **(A)** Nonembryogenic callus (NEC), **(B)** Embryogenic callus (EC), **(C)** Globular embryo( GE), **(D)** Cotyledon embryo (CE), bar=0.5 cm.

The mature seeds of fine single plants of *O. henryi* were collected in Mengguan town, Guiyang city in China (latitude: 26°14′23”N, longitude: 106°25′12″W) in Nov 2017. The authority responsible for the *O. henryi* resources is the Mengguan Forestry Station in Guizhou Province, China, who provided permission to collect the seeds of *O. henryi*. The formal identification of the plant material was undertaken by Prof. Mingtai An (Guizhou University). The matuer seeds were treated with concentrated H_2_S0_4_ for 30 min, washed with tap water for 30 min, disinfected in 75% alcohol for 1 min and then treated with 2% NaClO for 8 min, followed by 5 rinses in sterile distilled water. They were then soaked in sterile water for 24 h to make them swell and soften, and finally, mature embryos were obtained for somatic embryo induction. The somatic embryo induction methods referred to Wu et al., (2020). NEC (**Figure 1A**), EC (**Figure 1B**), GE (**Figure 1C**) and CE (**Figure 1D**) were collected. The culture medium on the surface of the calluses was washed with distilled water and placed into a 5 ml centrifuge tube, which was quickly frozen in liquid nitrogen and then transferred to a freezer at -80°C for storage. After the samples were collected, endogenous hormone determination and transcriptome sequencing were performed.

### Determination of endogenous hormones

The test samples were sent to Metware (http://www.metware.cn/). The contents of endogenous hormones, including AUX (indole-3-acetic acid, IAA), CKs (N6-isopentenyladenine, IP), trans-zeatin (tZ), cis-zeatin (cZ), dihydrozeatin (DZ), GAs (gibberellin, GA1, GA3, GA4, GA7, GA9, GA15, GA19, GA20, GA24, GA53) and abscisic acid (ABA), were detected by liquid chromatography-tandem mass spectrometry (LC−MS/MS).

### RNA extraction, library construction, and Illumina sequencing

Total RNA was extracted from NEC, EC, GE and CE using TRIzol reagent (Metvivre, Wuhan, China). RNA purity was examined using a NanoPhotometer spectrophotometer (Implen, CA, USA), RNA concentration was measured with a high-accuracy Qubit^®^ 2.0 fluorometer (Life Technologies, CA, USA), and RNA integrity was assessed using an Agilent 2100 Bioanalyzer (Agilent Technologies, CA, USA).

Poly A-tailed mRNA was enriched by Oligo(dT) magnetic beads, and then fragmentation buffer was added to break the RNA into short fragments. Using the short fragment RNA as a template, the first-strand cDNA was synthesized with six nucleobase random hexamers, buffer, dNTPs and DNA polymerase I to generate double-stranded cDNA, and then AMPure XP beads were used to purify the double-stranded cDNA. The purified double-stranded cDNA was then subjected to end repair, an A-tail was added, and a sequencing adapter was connected. Then, AMPure XP beads were used for fragment size selection, and finally, PCR enrichment was performed to obtain the final cDNA library.

After the library was constructed, Qubit 2.0 was used for preliminary quantification, and Agilent 2100 was used to detect the insert size of the library. The next experiment was performed only when the insert size met the expectation. Then, the effective concentration of the library was accurately quantified by the Q-PCR method (the effective concentration of the library was>2 nM) to complete the library inspection. Finally, the cDNA libraries were sequenced using the Novaseq 6000 platform (Illumina, San Diego, CA, USA).

### Assembly and gene function annotation

After obtaining sufficiently high-quality clean reads, Trinity software was used to splice clean reads to obtain unigenes for subsequent analysis. BLAST software was used to align the unigene sequences with the KEGG, NR, Swiss-Prot, GO, COG/KOG, and Trembl databases (E ≤ 1e-5), and HMMER software was used to compare them with the Pfam database to obtain annotation information for unigenes after predicting the amino acid sequences of unigenes.

### Differential gene analysis

FPKM (Fragments Per Kilobase of transcript per Million mapped reads) was used as an indicator to measure transcript or gene expression levels, and the threshold standard of DEGs was |log2Fold Change|≥1 and FDR < 0.05.

### Quantitative real-time PCR analysis

Six genes related to the regulation of endogenous hormones were selected for qRT−PCR verification, and the total RNA of the test samples was extracted by reverse transcription using a Thermo kit to obtain cDNA. A QIAGEN kit was used for qPCR detection. The specific reaction procedure was as follows: preheating at 95°C for 2 min, followed by 40 cycles at 95°C for 5 s and 60°C for 30 s. The relative expression level was calculated using the 2^-ΔΔCt^ method. HLM-Actin was used as the internal reference gene, and the primers are shown in **Table 1**. The relative expression levels were determined with 3 biological replicates, and each biological replicate was determined with 3 technical replicates.

**Table 1:** Primers for qRT−PCR.

Gene ID	Primer	Forward/Reverse primer	Amplicon length (bp)
Cluster-17474.55993	F	CAAGCGACATCGTTTCACCA	128
	R	CTTTCTCGGTAGTGTCATTGCTG	
Cluster-17474.166510	F	TGTTGGGAATGAAACTGAAGCA	151
	R	CCTCTTCCCACTCACTATCTCCA	
Cluster-17474.116314	F	TGGAGTTGTTGGAGTCCGATTT	105
	R	CAGCGAGAACCGAAAGGAGT	
Cluster-17474.121945	F	CATCAGAATACAGCAATTGGTTCC	186
	R	AGAAGCCTTCACCTGGTCAGC	
	R	GGCTCTGGCTATTCTGCTTTG	
Cluster-17474.122397	F	GATTTTCCACCTTCTGCGCTA	188
	R	GCTTCATCATCCCGTCGTTT	
Cluster-17474.27245	F	ATGGAGACTGTGGCGGTGAA	146
	R	TCTGGGTTTGTTGGATAACTGC	
HLM-18S (Cluster-17474.100110)	F	ATGCTTTCGCAGTTGTTCGTC	98

### Statistical analysis

All data were assessed for significant differences using Tukey’s test with SPSS 21.0 software. All data are presented as the mean ± standard deviation (SD) of three replicates. The graphs were created with Origin 9.0 and Photoshop 2019 software.

## Results

### Endogenous hormone contents

**Figure 2:**
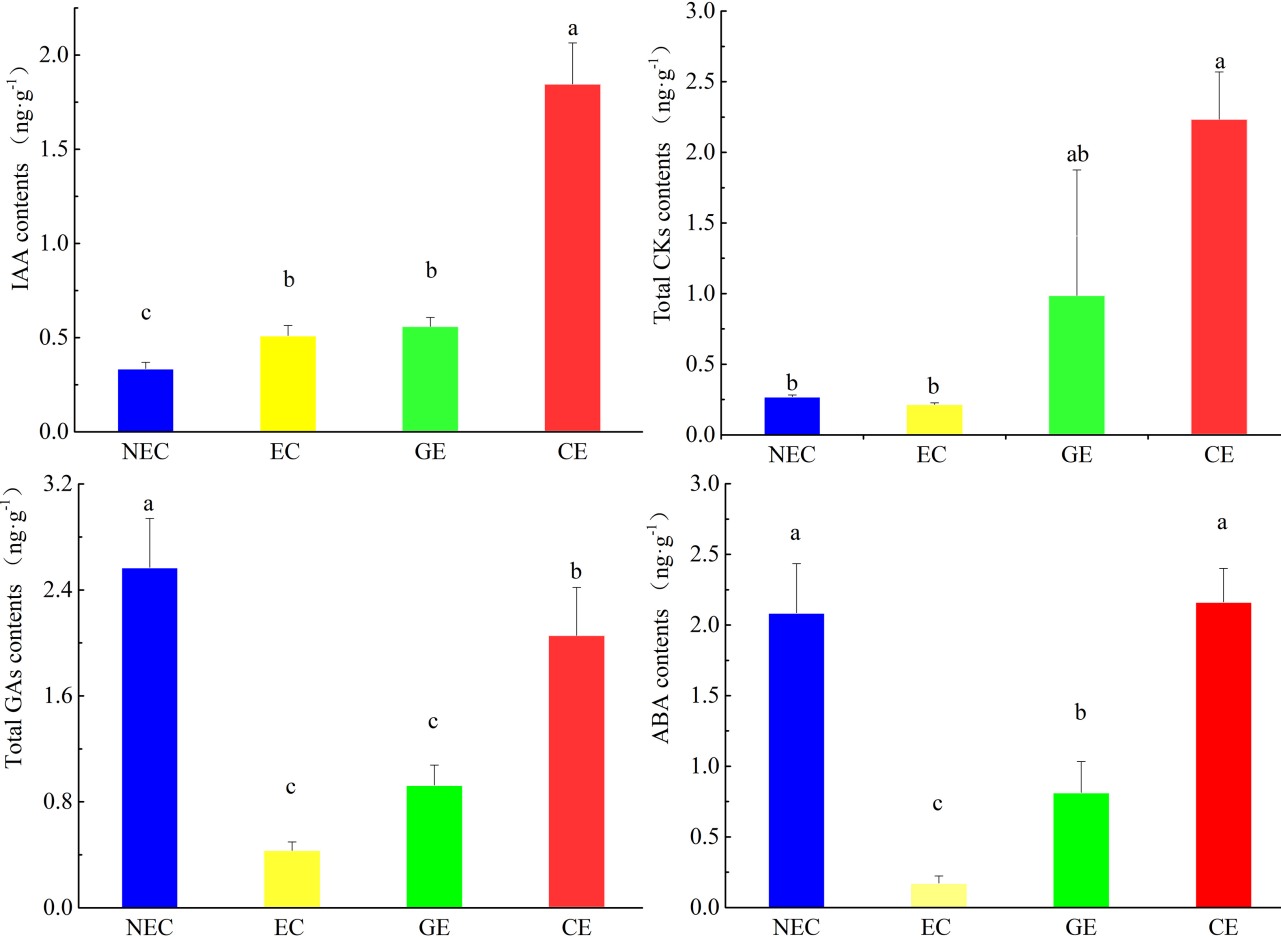
Contents of phytohormones at various phases of SE in *O. henryi*. Auxin is IAA, cytokinin content is the sum of IP, tZ, cZ and DZ, gibberellin content is the sum of GA1, GA3, GA4, GA7, GA9, GA15, GA19, GA20, GA24 and GA53, and abscisic acid is ABA. The different lowercase letters are significantly different using the Tukey’s test (P< 0.05).

There were significant differences in endogenous hormone contents at different SE stages of *O. henryi* (P<0.05, **Figure 2**). The content of IAA in NEC was the lowest, while the contents of CKs, GAs and ABA in EC were the lowest. The content of IAA in EC was higher than that in NEC. The contents of GAs and ABA in NEC were 5.5 and 12 times those in EC, respectively. The results showed that relatively high IAA and low CKs, GAs and ABA contents promoted EC formation. With EC development, the contents of IAA, CKs, GAs and ABA gradually increased and were the highest in CE, which were 3.7, 10.4, 10.7 and 12.7 times those in EC, respectively. These results indicated that these four endogenous hormones contents were increased with somatic embryo development in *O. henryi*, and each tissue stage had its specific hormone content.

### RNA sequence analysis and quantitative real-time PCR

**Figure 3:**
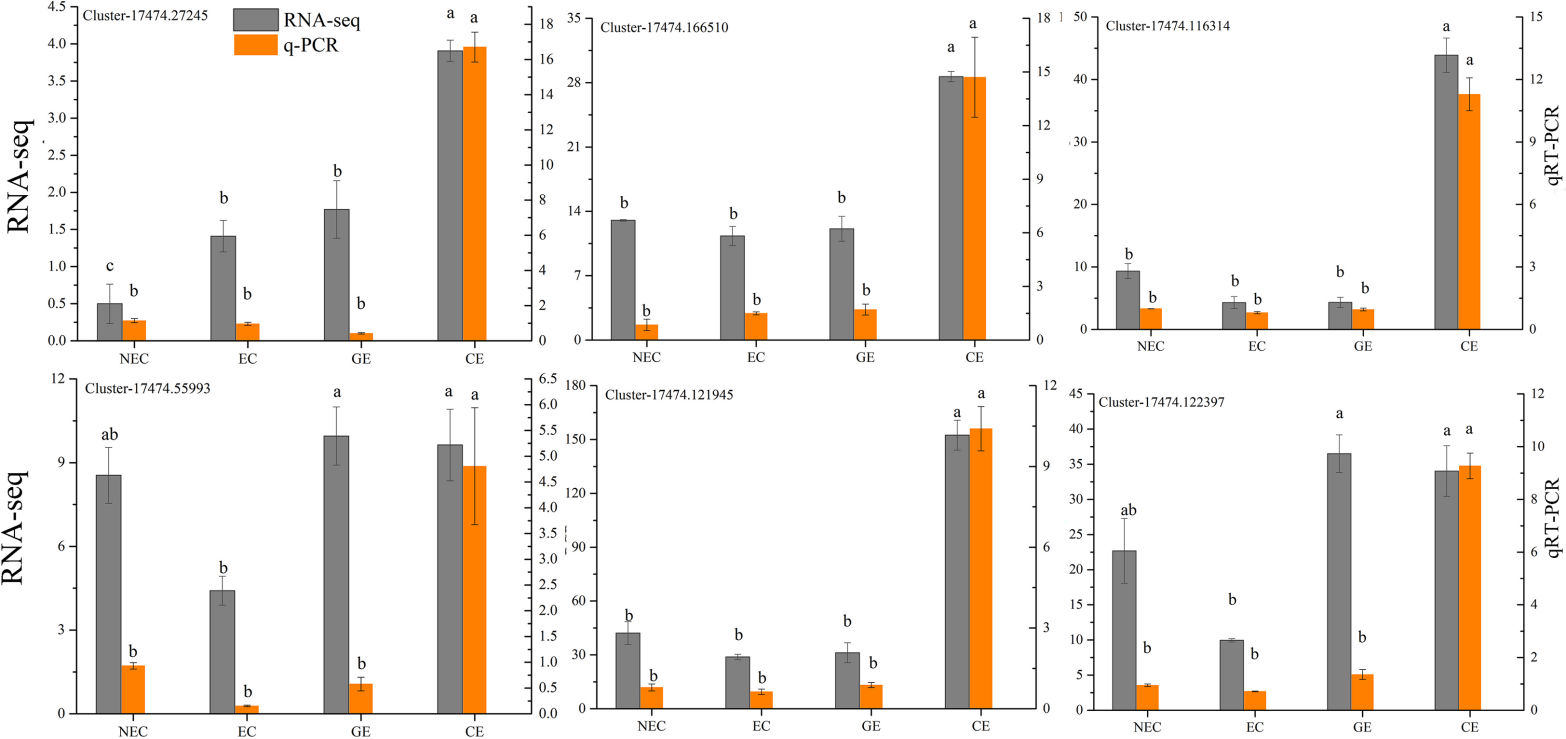
Analysis of the RNA-Seq and qRT−PCR data for the 6 genes. The different lowercase letters are significantly different using the Tukey’s test (P< 0.05).

In this study, a total of 12 samples were sequenced, and 105.67 GB of clean data was obtained. The clean data, Q30, Q20, GC, clean reads and transcripts of each sample are shown in **Table 2**. The sequence and assembly quality levels of the 12 samples were high. The overall expression trends of the qRT−PCR and RNA-Seq expression levels of the 6 genes were consistent (**Figure 3**), indicating that the transcriptome data were reliable and could be further analysed.

**Table 2:** Transcriptome sequence analysis of 12 samples from different SE stages of *O. henryi*.

Sample	Raw Reads	Clean Reads	Clean Base (G)	Q20 (%)	Q30 (%)	GC Content (%)
NEC1	61525848	59981196	9	98.19	94.36	44.46
NEC2	50257358	49093036	7.36	98.19	94.37	44.91
NEC3	58819680	57217534	8.58	98.27	94.54	44.46
EC1	62388836	60599088	9.09	98.22	94.44	44.72
EC2	56115266	54671970	8.2	98.3	94.64	44.51
EC3	59556858	58190132	8.73	98.15	94.21	44.6
GE1	56381688	54866444	8.23	98.26	94.56	44.81
GE2	57158306	55918886	8.39	98.31	94.65	44.98
GE3	64227500	62546844	9.38	98.29	94.61	44.94
CE1	64559336	62809376	9.42	98.28	94.59	44.72
CE2	73366476	71246432	10.69	98.27	94.58	44.64
CE3	59086514	57301864	8.6	98.29	94.61	44.72

### Annotation and functional classification of unigenes

The functions of unigenes were annotated by BLAST software and were predicted by comparative analysis with seven databases (KEGG, NR, Swiss-Prot, Trembl, KOG, GO and Pfam). Among the 235394 unigenes distributed to each of the databases, 47.93%, 59.47%, 39.58%, 59.05%, 35.75%, 49.27% and 40.44% of the genes were annotated (**Additional file 1**). The highest species match was found in *Lupinus angustifolius* (18.74%), followed by *Cajanus cajan* (17.21%) and *Glycine max* (4.23%) (**Additional file 2**).

A total of 115981 genes were annotated in the GO database, among which there were a large number of genes in cell (76165), cell component (76047), organelle (59197), cell membrane (36686) and organelle component (27642) for the cellular component; binding activity (72917) and catalytic activity (61800) for molecular function; and cellular process (73879) and metabolic process (64693) for biological process (**Additional file 3**).

A total of 84165 genes were annotated in the KOG database, and a total of 25 classification functions were obtained. The top three KOG functions were “General function prediction only” (28881), “Posttranslational modification, protein turnover, chaperones” (7702) and “Signal transduction mechanisms” (7492), and the lowest was “Cell motility” (37) (**Additional file 4**).

In total, 112816 unigenes were mapped into 141 KEGG database pathways. The top eleven KEGG pathways were “Metabolic pathways” (20565), “Biosynthesis of secondary metabolites” (9661), “Carbon metabolism” (2930), “Protein processing in endoplasmic reticulum” (2296), “RNA transport” (2283), “Plant−pathogen interaction” (2232), “Biosynthesis of amino acids” (2207), “Amino sugar and nucleotide sugar metabolism” (2169), “Endocytosis” (2151), “Plant hormone signal transduction” (2018) and “Starch and sucrose metabolism” (1961). In summary, the annotated gene information could provide data support for exploring its biological functions and revealing the molecular mechanism involved in SE at different stages for *O. henryi* (**Additional file 5**).

### Analysis of DEGs and KEGG metabolic pathways

**Figure 4:**
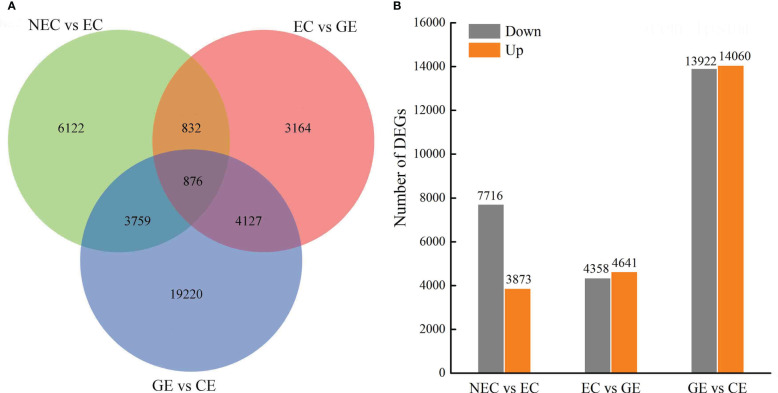
DEG numbers at SE stages in O. henryi. **(A)** Venn diagram show the DEGs in NEC vs EC, EC vs GE, and GE vs. CE; **(B)** Statistic of Up/Down regulated genes in pairwise comparisons of NEC vs EC, EC vs GE, and GE vs. CE.

The numbers of DEGs in NEC vs. EC, EC vs. GE, and GE vs. CE were 11589, 8999, and 27982, respectively, totalling 38100, and the number of common DEGs was 876 (**Figure 4A**). In NEC vs. EC, 7716 DEGs were downregulated and 3873 were upregulated, of which the number of upregulated DEGs was 2 times that of downregulated DEGs. In EC vs. GE, 4358 DEGs were downregulated and 4641 were upregulated. GE vs. CE had the largest number of DEGs, with 14060 upregulated and 13922 downregulated (**Figure 4B**).

**Figure 5:**
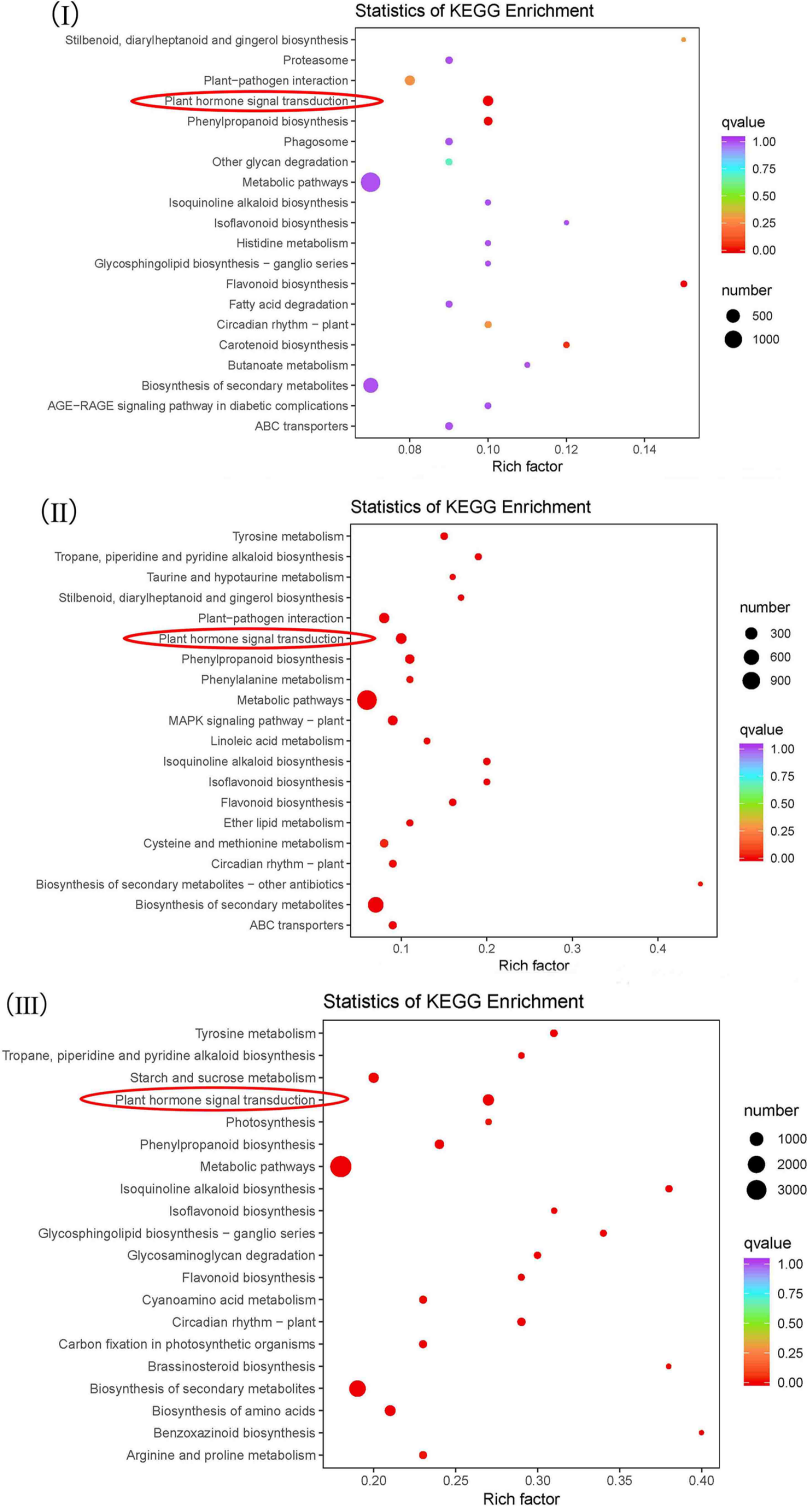
KEGG enrichment analysis of the differential genes. I-III represent KEGG enrichment of differential genes at NEC vs. EC, EC vs. GE and GE vs. CE, respectively.

The DEGs of NEC vs. EC, EC vs. GE and GE vs. CE were annotated into the KEGG database to obtain 137, 135 and 141 metabolic pathways, respectively (**Additional file 6**). Among them, metabolic pathways such as “plant hormone signal transduction”, “metabolic pathways”, “biosynthesis of secondary metabolites”, “circadian rhythm - plant”, “flavonoid biosynthesis”, “isoflavonoid biosynthesis”, “isoquinoline alkaloid biosynthesis” and “phenylpropanoid biosynthesis” were significantly enriched (**Figure 5**).

The above analysis showed that there were significant differences in the contents of endogenous hormones at different SE stages. To understand how the changes in endogenous hormone contents were regulated by the gene expression of the plant hormone biosynthesis and signal transduction pathways, we focused on five metabolic pathways involved in the biosynthesis and signal transduction of auxin, cytokinin, gibberellin and abscisic acid. The results showed that (1) tryptophan metabolism (ko00380) was annotated with 42 DEGs; (2) zeatin biosynthesis (KO00908) was annotated to 27 DEGs; (3) diterpenoid biosynthesis (KO00904) was annotated to 24 DEGs; (4) carotenoid biosynthesis (KO00906) was annotated to 48 DEGs; and (5) in plant hormone signal transduction (KO04075), there were 192, 90, 97 and 69 differentially expressed signal transduction genes annotated with auxin, cytokinin, GAs and abscisic acid, respectively (**Additional file 7**). The results showed that the differences in endogenous hormone contents at different SE stages were regulated by related genes encoding plant hormones.

I-III represent KEGG enrichment of differential genes at NEC vs. EC, EC vs. GE and GE vs. CE, respectively.

### DEGs related to phytohormones regulating SE in *Ormosia henryi*

**Figure 6:**
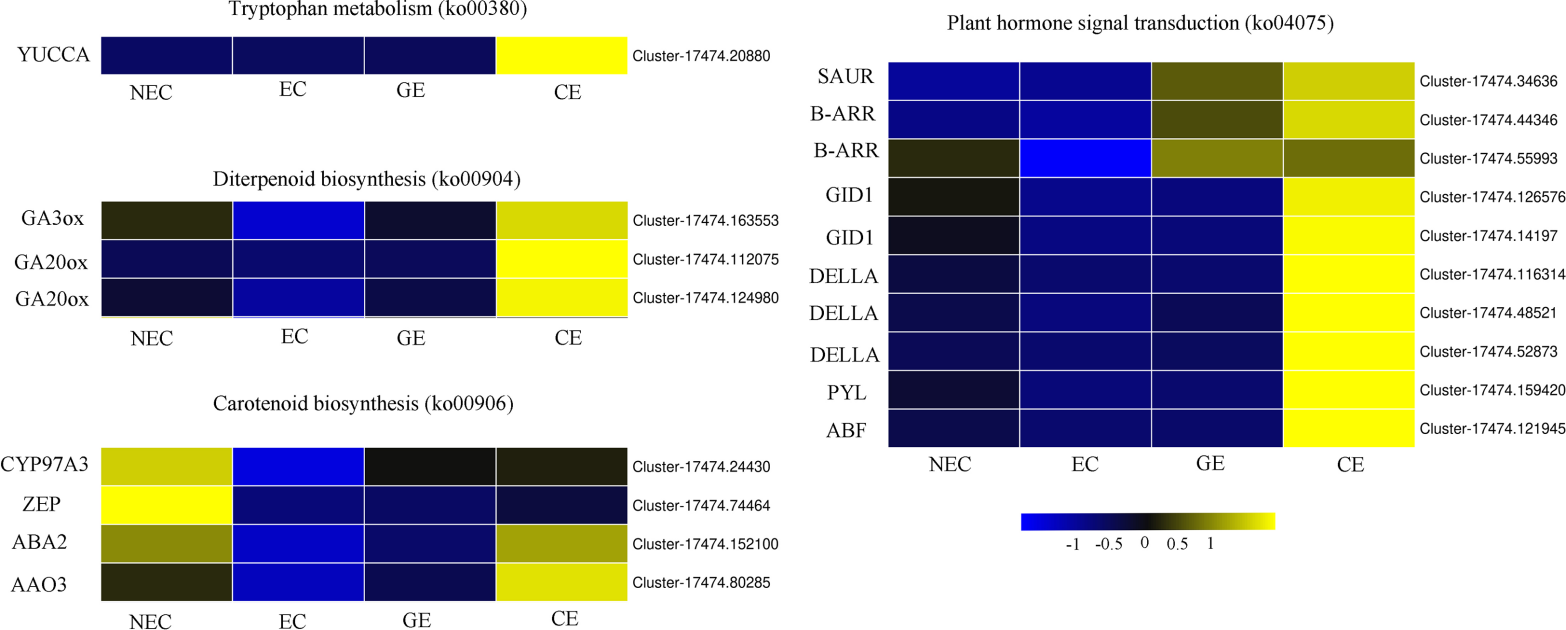
Differentially expressed genes of endogenous hormone biosynthesis and signal transduction in *O. henryi*.

To further explore the related genes that affect the changes in endogenous hormones during SE for *O. henryi*, DEGs related to plant hormone biosynthesis and signal transduction were screened from the transcriptome data, as shown in **Figure 6**. In tryptophan metabolism (ko00380), there was a DEG, *YUCCA*, that was not expressed in NEC, was expressed at low levels in EC and GE, and was expressed at high levels in CE. In diterpene biosynthesis (ko00904), one *GA3ox* and two *GA20ox* genes were highly expressed in NEC and low in EC, and these expression levels gradually increased with EC development. In carotenoid biosynthesis (ko00906), the expression patterns of *CYP97A3* (cytochrome P450 enzymes), *ZEP* (zeaxanthin epoxidase), *ABA2* and *AAO3* (Arabidopsis aldehyde oxidase 3) were the same as those of *GA3ox* and two *GA20ox* genes during SE for *O. henryi*. In plant hormone signal transduction (ko04075), 1 small auxin-up RNA (*SAUR*), 2 type-B ARA-bidopsis response regulators (*B-ARR*), 2 GA-insensitive dwarf 1 (*GID1*), 3 *DELLA*, 1 PYR1-like (*PYL*) and 1 ABRE-binding factor (*ABF*) were screened. Their expression patterns were consistent with the trend of endogenous hormone contents in SE at different stages for *O. henryi*. It was speculated that these genes were involved in the biosynthesis of corresponding endogenous hormones and affected the SE process of *O. henryi*.

### TFs related to phytohormones regulating SE in *Ormosia henryi*

**Figure 7:**
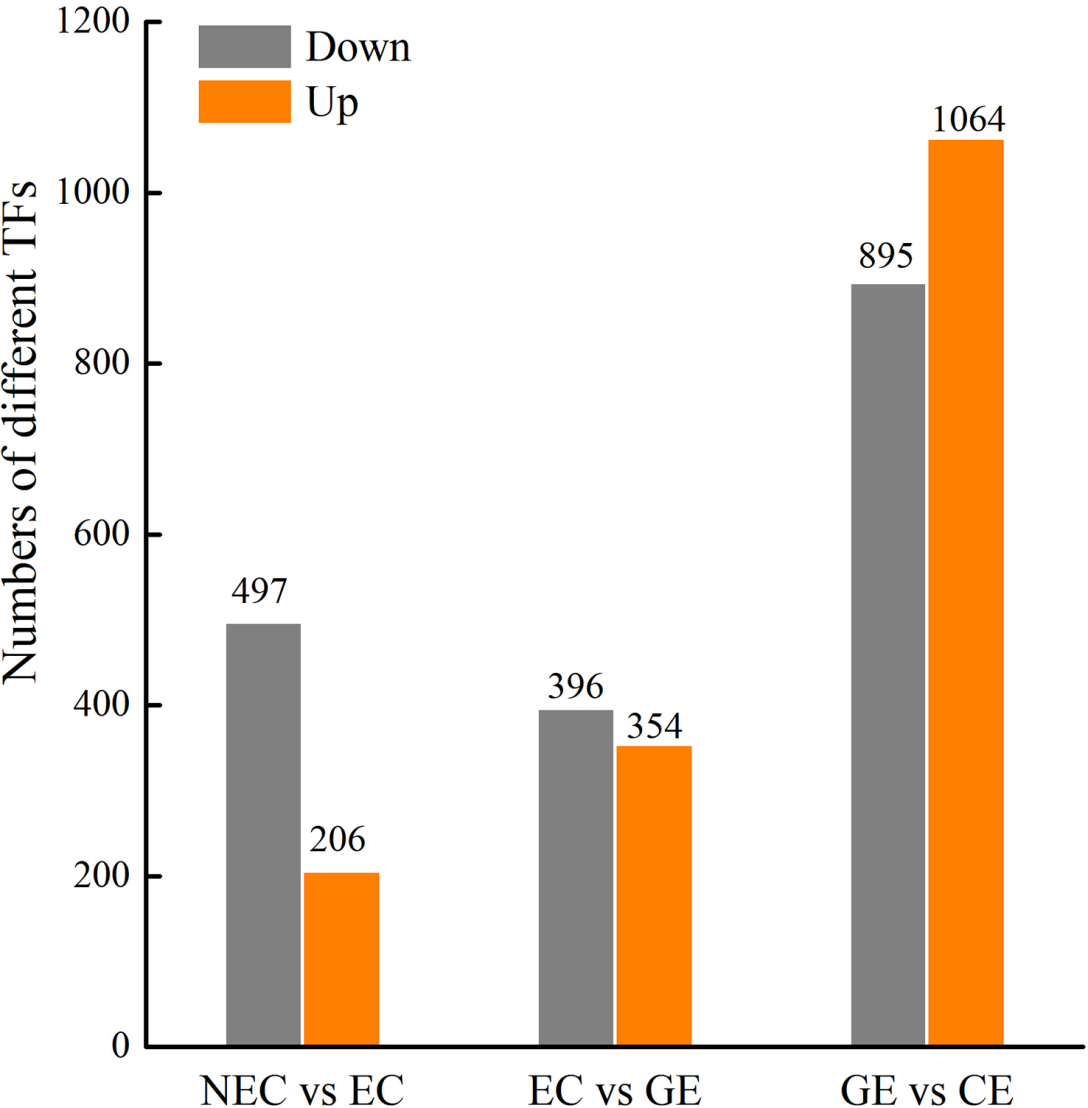
Different TFs at SE stages in *O. henryi*.

**Figure 8:**
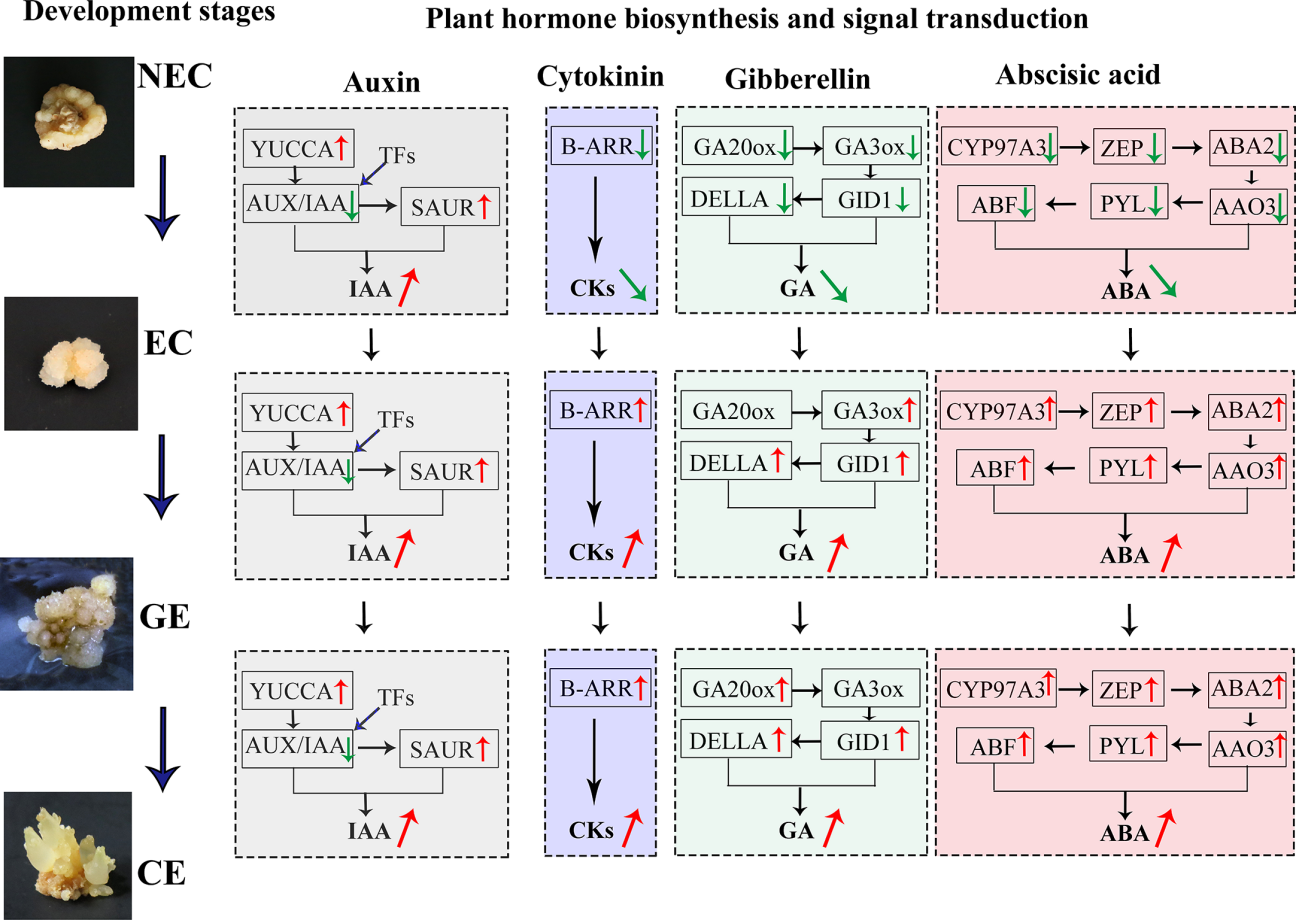
DGEs and TFs of endogenous hormone biosynthesis and signal transduction regulated SE process at different stages in *O. henryi*.

A large number of differential TFs were found during SE of *O. henryi*, with 713 in NEC vs. EC, 750 in EC vs. GE, and 1959 in GE vs. CE (**Figure 7**). Among them, there were 66, 62 and 188 differential TFs involved in plant hormone regulation, respectively, 55 *AUX/IAAs* and 58 *ARFs* in auxin; 8 *CER1s*, 30 *B-ARRs* and 5 *A-ARRs* in cytokinin; 35 *DELLAs* and 32 TFs in gibberellin; and 15 ABFs in abscisic acid, for a total number of 238 differential TFs during SE for *O. henryi* (**Additional file 8**). It is worth noting that most *AUX/IAA* TFs were downregulated during EC formation and GE differentiation into CE and highly expressed in NEC, while other TFs were upregulated and downregulated at different SE stages. These results suggest that *AUX/IAA* played important roles in EC formation and CE differentiation of *O. henryi* (**Figure 8**), and the regulation of its development by other TFs is a complex process.

## Discussion

Plant hormones regulate various processes of plant growth, development and environmental adaptation. They both independently and cooperatively regulate plant seed germination, vegetative growth, reproductive growth, embryonic development, seed maturation and dormancy and other growth and development processes, as well as the adaptation of biotic and abiotic environmental stress during the plant growth cycle (Li and Li, 2019). Among them, IAA is an important hormone regulating SE development. The content of IAA in EC of *O. henryi* was higher than that in NEC and increased significantly as the EC developed into GE and CE, which was similar to the findings for *Cyathea delgadii* (Grzyb et al., 2017), *Norway Spruce* (Vondrakova et al., 2018), and *Medicago truncatula* (Kępczyńska and Orłowska, 2021) larch (Von Aderkas et al., 2001), these results indicated that the increase in auxin concentration was related to the establishment of embryo polarity, the initiation of stem cells, shoot apical meristem maintenance (Vondrakova et al., 2018), and stimulated *M. truncatula MtSERK1* gene expression (Nolan et al., 2003), which has been associated with SE and *in vitro* root formation in several plant species (Nolan et al., 2009). Chen et al. (2020) reported that *YUCCA*, *NIT*, and *CYP71A13* genes involved in auxin synthesis were upregulated from NEC to EC in longan, and *YUCCA* was upregulated in the early somatic embryo. In this study, *YUCCA* and *SAUR* genes involved in auxin biosynthesis and signal transduction were downregulated in NEC and were gradually upregulated with EC development. It was speculated that the differential expression of *YUCCA* and *SAUR* affected the *IAA* level, thereby playing a key role in regulating the SE process in *O. henryi*.

The CKs content of EC was lower than that of NEC in *O. henryi*, which was consistent with the findings of Pérez-Jiménez et al. (2013). The study of Fraga et al. (2016) similarly supported this view and reported that the decrease in CKs levels was associated with embryogenic potential. *B-ARRs*, as positive factors and key genes of cytokinin transduction, mediate cytokinin signal activation, upregulate the expression of the downstream gene *CYCLIND3*, and promote cells to re-enter the cell cycle (Ikeuchi et al., 2019), playing important roles in callus induction and stem cell regulation (Ikeuchi et al., 2013). *B-ARR* was upregulated with EC development, and the expression trend was consistent with CKs levels, indicating that the differential expression of *B-ARR* affected CKs levels in the SE process for *O. henryi*, thus regulating EC induction and differentiation and cotyledon embryo germination. Similar results have been reported in SE for *G. hirsutum* (Cheng, 2016).

GAs and ABA play important roles in regulating plant development, adapting to stress responses and improving crop yield (Khan et al., 2012; Li and Li, 2019). It was surprising to find that low ABA : GAs ratios in *M. truncatula* acted synergistically to stimulate SE, however, it is a high ABA : GAs ratio in Arabidopsis (Nolan et al., 2014), these have utility in inducing and improving SE for regeneration in different plant. However, the contents of GAs and ABA in EC were significantly lower than those in NEC of *O. henryi* and increased gradually with EC development. Similar results were reported in *Hevea brasiliensis* (Etienne et al., 1993), *Norway Spruce* (Vondrakova et al., 2018) and tree fern (Grzyb et al., 2017). *GA3ox* and *GA20ox* are the rate-limiting enzymes in the biosynthesis of GAs, and *GA20ox* can oxidize inactive GA53 and GA12 into GA9 and GA20 with biological activity. *GA3ox* catalyses the synthesis of GA1, GA1, GA3 and GA4 with GA9 and GA20 as substrates (Yamaguchi, 2008). Zheng et al. (2016) demonstrated interaction between AUX and GAs in the promotion of SE and document an inverse correlation between bioactive GAs and SE in soybean, the increase of GA2ox expression regulated negatively SE in Arabidopsis (Nolan et al., 2014). Mitsuhashi et al. (2003) reported that *GA20ox* was continuously expressed during embryo development in carrots, and *GA3ox* was continuously upregulated after embryonic developmental induction. The gibberellin receptor GID1 and the signal transduction gene *DELLA* also showed the same expression trends, and their expression patterns were consistent with GAs levels, indicating that their differential expression affected GAs biosynthesis in the SE process for *O. henryi*.

ABA content was more higher in NEC and CE, indicating that ABA could inhibite EC induction and promote CE maturation and differentiation. Similar results were shown in *M. truncatula* (Kępczyńska and Orłowska, 2021), while Pérez-Jiménez et al. (2013) found no significant difference in ABA content between NEC and EC *Prunus persica.* Perhaps these differences between NEC and EC are due to the different content of ABA catabolites and conjugates. The ABA biosynthesis genes *CYP97A3*, *ZEP*, *ABA2* and *AAO3* were expressed at low levels in EC for *O. henryi* and at high levels in GE differentiating into CE. Similar results were also found in SE development for *G. hirsutum* (Cheng, 2016). As an ABA receptor protein, *PYL* was downregulated in dedifferentiated tissues of eucalyptus (Xiao et al., 2020). Jie (2016) reported that *PYL* was significantly upregulated during SE for *G. hirsutum*, which was consistent with the results of this study. These results provide a reliable molecular basis for explaining why high contents of GAs and ABA can inhibit SE induction but promote SE maturation and differentiation in *O. henryi*.

TFs play a key role in plant growth and development. They are widely involved in not only plant growth and development but also stress response and hormone regulation. It was found that *AUX/IAA* TFs involved in the SE process of *O. henryi* were highly expressed in NEC and were downregulated in EC formation and GE differentiation into CE. Similar results were reported in the SE process for *G. hirsutum* (Yang et al., 2012; Cheng, 2016). Quintana-Escobar et al. (2019) found that the expression of *AUX/IAA* decreased gradually with the SE process in coffee. It was speculated that the high expression of *AUX/IAA* promoted NEC formation and inhibited EC formation and CE differentiation in *O. henryi*. To explain the important role of *AUX/IAA* in SE development, previous studies have shown that *AUX/IAA* proteins form a dimer with ARF at low auxin levels and inhibit ARF activity by binding to TPL coinhibitory factor (TOPLESS), thereby inhibiting the response of auxin signal transduction genes. In contrast, *AUX/IAA-ARF* was degraded by *SCFTIR1/AFB* at high auxin levels and activated *ARF*, thereby positively or negatively regulating the response of downstream auxin signals (Zheng et al., 2014; Quintana-Escobar et al., 2019). In general, exogenous auxin was the key hormone for EC induction. The high auxin content could stimulate the expression of the *AUX/IAA* gene in EC induction medium and promote NEC formation in *O. henryi*, resulting in a low EC induction rate, explaining why the NEC induction rate was high and the EC induction rate was low in the process of SE induction for *O. henryi*. The authors considered that the EC induction rate could be increased by using other hormones instead of high auxin concentrations or adjusting medium components. In the differentiation stage of somatic embryos, the auxin content was low in the medium, the *AUX/IAA-ARF* dimer was formed and *AUX/IAA* expression was inhibited, providing a reliable theoretical basis for the downregulation of the *AUX/IAA* gene in CE differentiation for *O. henryi*. Other TFs involved in the regulation of plant hormones during SE were upregulated and downregulated, indicating that these TFs regulated SE in *O. henryi* as part of a complex process.

## Conclusion

The changes in endogenous hormone levels were the material basis for the growth and morphogenesis of SE in *O. henryi*, and the related expression profiles of genes involved in plant hormone biosynthesis and signal transduction affected the endogenous hormone levels, thus regulating the SE process. Relatively high IAA and low CKs, GAs and ABA contents promoted EC formation. The differential expression of auxin biosynthesis and signal transduction genes (*YUCCA, SAUR*), cytokinin (*B-ARR*), gibberellin (*GA3ox*, *GA20ox*, *GID1* and *DELLA*) and abscisic acid (*ZEP*, *ABA2*, *AAO3*, *CYP97A3*, *PYL* and *ABF*) affected the endogenous hormone levels at different SE stages. The downregulated expression of *AUX/IAA* and differential TFs inhibited NEC induction and promoted EC formation and GE differentiation into CE. Other TFs involved in the regulation of plant hormones during SE were upregulated and downregulated, providing a technical and theoretical basis for further mining its mechanism.

## Data availability statement

The datasets presented in this study can be found in online repositories. The names of the repository/repositories and accession number(s) can be found in the article/**Supplementary Material**.

## Author contributions

The project was conceived by GYW and XLW. The experiment was executed by GYW, XW and YW. All coauthors drafted, reviewed, and edited the manuscript. All authors read and approved the final version of this manuscript.

## Supplementary material

Additional file 1 Gene annotation information.

Additional file 2 Species match.

Additional file 3 GO database.

Additional file 4 KOG database.

Additional file 5 KEGG database pathways.

Additional file 6 KEGG enrichment analysis of the differential genes.

Additional file 7 The DEGs analysis of plant hormone biosynthesis pathway and signal transduction pathway.

Additional file 8 The number of differential TFs for phytohormones.
