# Supplementary figures and images for: Changes and transcriptome regulation of endogenous hormones during somatic embryogenesis in Ormosia henryi Prain
Source: Front Plant Sci. 2023 Apr 3;14:1121259. doi: 10.3389/fpls.2023.1121259 (PMC10106752; doi:10.3389/fpls.2023.1121259)

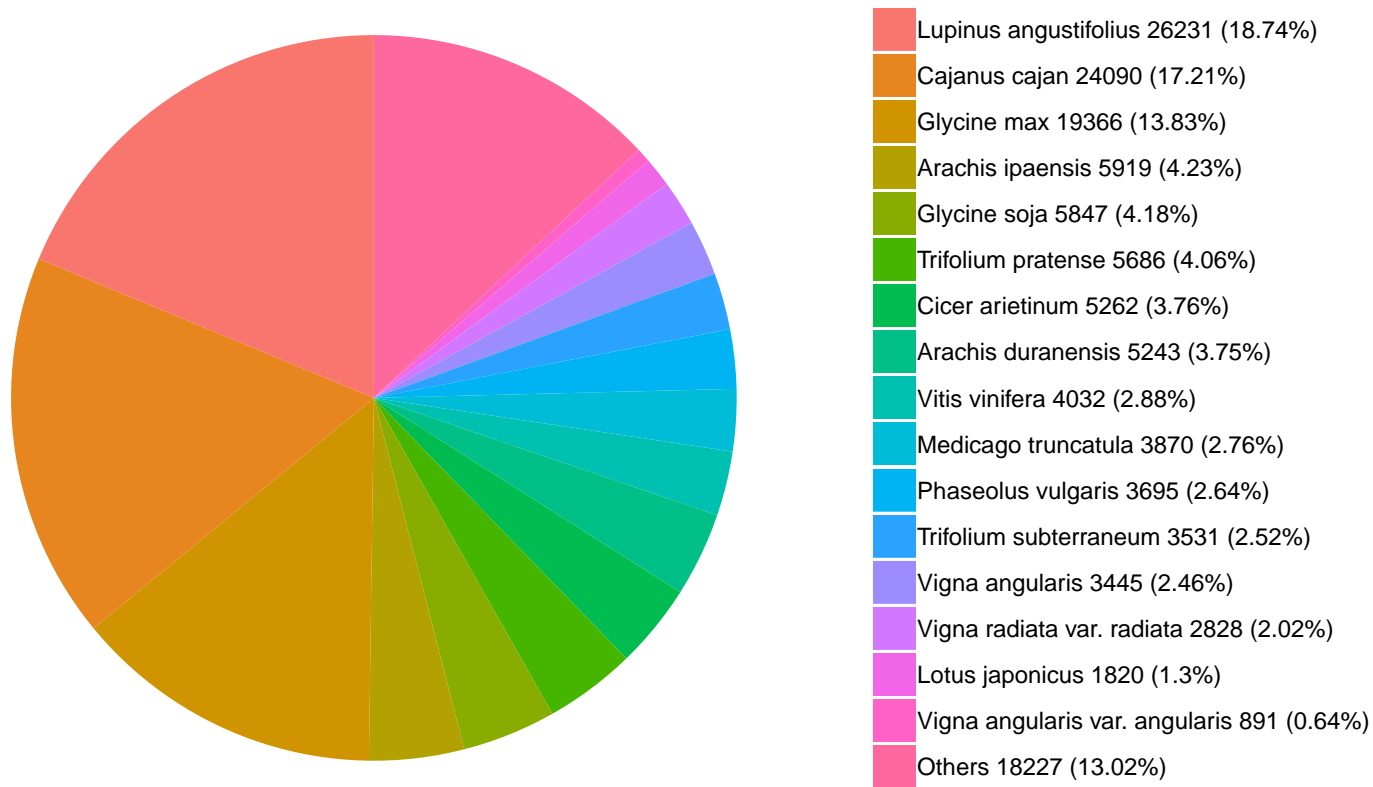

Supplement: Additional file 7 — The DEGs analysis of plant hormone biosynthesis pathway and signal transduction pathway. [file DataSheet_1.pdf]
